# Expression and Sequence Variants of Inflammatory Genes; Effects on Plasma Inflammation Biomarkers Following a 6-Week Supplementation with Fish Oil

**DOI:** 10.3390/ijms17030375

**Published:** 2016-03-15

**Authors:** Hubert Cormier, Iwona Rudkowska, Simone Lemieux, Patrick Couture, Marie-Claude Vohl

**Affiliations:** 1 Institute of Nutrition and Functional Foods (INAF), Laval University, Quebec City, QC G1V 0A6, Canada; hubert.cormier.1@ulaval.ca (H.C.); simone.lemieux@fsaa.ulaval.ca (S.L.); patrick.couture@crchudequebec.ulaval.ca (P.C.); 2 School of Nutrition, Laval University, Quebec City, QC G1V 0A6, Canada; 3 Endocrinology and Nephrology, Department of Kinesiology, CHU de Québec Research Center, Laval University, Quebec City, QC G1V 0A6, Canada; iwona.rudkowska@crchudequebec.ulaval.ca; 4 Centre Hospitalier Universitaire (CHU) de Québec Research Center, Laval University, Quebec City, QC G1V 4G2, Canada

**Keywords:** omega-3 polyunsaturated fatty acids, polymorphism, inflammation, nutrigenetics

## Abstract

(1) Background: A growing body of literature suggest that polymorphisms (SNPs) from inflammation-related genes could possibly play a role in cytokine production and then interact with dietary *n*-3 fatty acids (FAs) to modulate inflammation. The aim of the present study was to test whether gene expression of selected inflammatory genes was altered following an *n*-3 PUFA supplementation and to test for gene–diet interactions modulating plasma inflammatory biomarker levels. (2) Methods: 191 subjects completed a 6-week *n*-3 FA supplementation with 5 g/day of fish oil. Gene expression of *TNF*-α and *IL6* was assessed in peripheral blood mononuclear cells (PBMCs) using the TaqMan technology. Genotyping of 20 SNPs from the *TNF*-*LTA* gene cluster, *IL1*β, *IL6* and *CRP* genes was performed. (3) Results: There was no significant reduction of plasma IL-6, TNF-α and C-reactive protein (CRP) levels after the 6-week fish oil supplementation. *TNF*-α and *IL6* were slightly overexpressed in PBMCs after the supplementation (fold changes of 1.05 ± 0.38 and 1.18 ± 0.49, respectively (*n* = 191)), but relative quantification (RQ) within the −0.5 to 2.0 fold are considered as nonbiologically significant. In a MIXED model for repeated measures adjusted for the effects of age, sex and BMI, gene by supplementation interaction effects were observed for rs1143627, rs16944, rs1800797, and rs2069840 on IL6 levels, for rs2229094 on TNF-α levels and for rs1800629 on CRP levels (*p* < 0.05 for all). (4) Conclusions: This study shows that a 6-week *n*-3 FA supplementation with 5 g/day of fish oil did not alter gene expression levels of *TNF*-α and *IL6* in PBMCs and did not have an impact on inflammatory biomarker levels. However, gene–diet interactions were observed between SNPs within inflammation-related genes modulating plasma inflammatory biomarker levels.

## 1. Introduction

Evidence suggests that fatty acids (FAs) can modulate adipokine production, thus accelerating and influencing an individual’s inflammatory response [1]. Omega-3 (*n*-3) FAs have potent anti-inflammatory effects. *n*-3 FAs may affect inflammatory processes through modulation of eicosanoid metabolism and by regulating transcription factors genes involved in inflammation [2]. The resolution of inflammation involves active biochemical compounds, termed resolvins and protectins, which enable inflamed tissues to restore homeostasis [3]. However, these effects have been demonstrated in *in vitro* studies [4,5]. The extent of these effects *in vivo* is not clearly established and remains unclear. Dietary FAs, in particular saturated FAs in addition to *n*-3 and omega-6 FAs could potentially modulate the expression of genes encoding cytokines, possibly altering plasma cytokine levels as well [6].

A review by Joffe *et al.* suggested that polymorphisms (SNPs) within inflammation-related genes may interact with environmental factors, such as dietary intakes, to modulate an individual’s susceptibility to develop obesity and its comorbidities [6]. Interaction effects between dietary FAs and variations in inflammation-related genes such as *tumor necrosis factor* α (*TNF*-α) and *interleukin 6* (*IL6*) may influence obesity phenotypes [6]. A growing body of literature suggest that SNPs from inflammation-related genes could possibly play a role in cytokine production and then interact with dietary *n*-3 FAs to modulate inflammation [7].

*n*-3 FAs are beneficial for inflammation-related diseases such as rheumatoid arthritis, inflammatory bowel diseases and asthma [8]. Accordingly, marine *n*-3 FAs have been shown to decrease expression levels of inflammation-related genes as well as plasma concentrations of cytokine and C-reactive protein (CRP) [6]. However, there are no extensive studies showing a strong relationship between expression of inflammation-related genes and *n*-3 FA supplementation. Previous research from our laboratory has demonstrated changes in inflammatory pathways in human peripheral blood mononuclear cells (PBMCs) after an *n*-3 FA supplementation via a transcriptomic approach [9]. Similarly, Bouwens *et al.* (2009), have examined the effects of high doses of *n*-3 FA supplementation on whole-genome gene expression profiles in PBMCs, and reported a decrease in expression of genes involved in inflammatory- and atherogenic-related pathways. Although, according to their results, *TNF*-α and *IL6* gene expression levels were not underexpressed after the supplementation [10].

The aim of the present study was to test whether gene expression of inflammation-related genes is altered following an *n*-3 FA supplementation and to test for possible gene–diet interactions with SNPs within these genes modulating plasma inflammatory biomarker levels. We hypothesized that a 6-week marine *n*-3 FA supplementation decreases gene expression of selected inflammation-related genes and also decreases plasma levels of inflammatory biomarkers such as CRP, TNF-α and IL-6 under the influence of SNPs within inflammation-related genes.

## 2. Results

Population characteristics have been previously described here [11] for the total cohort and here [12] for this specific population included in this reanalysis. Briefly, participants have a higher BMI following the 6-week *n*-3 FA supplementation (*p* = 0.006), but the absolute difference remains extremely low at 0.1 kg/m^2^, with no effects on waist circumference. After the supplementation, TG levels decreased, as expected (*p* < 0.0001). Table 1 reports the descriptive characteristics of study participants at baseline.

Pre- and post-supplementation inflammatory marker levels are presented in Table 2. There was no significant difference observed in inflammatory marker levels. However, a large inter-individual variability was observed in the inflammatory response to a fish oil supplementation. For instance, 45.0%, 47.6% and 48.2% of study participants increased their plasma levels of CRP, TNF-α and IL-6 respectively after the 6-week supplementation.

Energy intakes were lower after the *n*-3 FA supplementation (*p* = 0.003) as shown in Table 3. When looking at macronutrient distribution, there was a shift towards an increase in total fat together with a decrease in carbohydrates (absolute difference of 24.8 g/day) and proteins (absolute difference of 5.4 g/day). In fat intake, PUFA intakes were higher (*p* = 0.001) as a result of taking 5 g/day of fish oil supplements while saturated FA intakes were lower with an absolute difference of 3.7 g/day.

**Figure 1:**
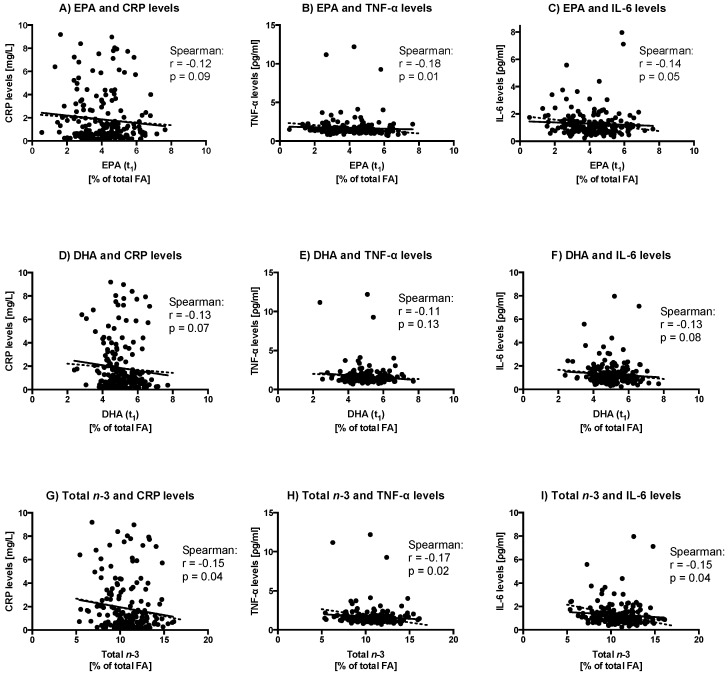
Partial Spearman correlations between EPA, DHA or total *n*-3 PUFA (in % of total FA) from phospholipids and inflammatory marker levels controlled for baseline data (both FAs and inflammatory marker levels), age, sex and BMI. Panels (**A**–**C**) show correlations between EPA (in % of total FA) and inflammatory markers; Panels (**D**–**F**) show correlations between DHA (in % of total FA) and inflammatory markers; and panels (**G**–**I**) show correlations between total *n*-3 (in % of total FA) and inflammatory markers; straight lines: unadjusted regression slope, and dotted lines: adjusted regression slope with baseline inflammatory marker levels, baseline *n*-3 from phospholipids levels, age, sex and BMI (*n* = 191).

Partial Spearman correlations between EPA, DHA or total *n*-3 FA levels (in % of total FA) from plasma phospholipids and inflammatory marker levels adjusted for baseline data (both FAs and inflammatory marker levels), age, sex and BMI are presented in Figure 1. Briefly, total plasma *n*-*3* FA levels negatively correlates with CRP (*r* = −0.15, *p* = 0.04), TNF-α (*r* = −0.17, *p* = 0.02), and IL-6 levels (*r* = −0.15, *p* = 0.04). Looking only at plasma EPA levels, a negative correlation was found with TNF-α levels (*r* = −0.18, *p* = 0.01) while trends were observed with CRP and IL-6 levels. Plasma DHA levels tended to be negatively correlated with CRP and IL-6 levels (*p* < 0.10, for all).

**Figure 2:**
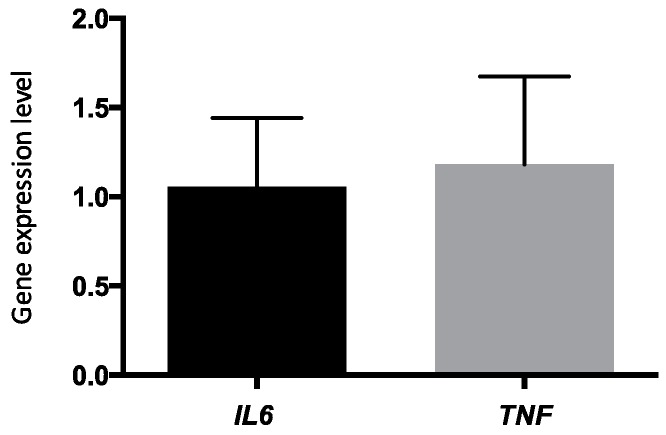
*IL6* and *TNF*-α gene expressions after a 6-week *n*-3 FA supplementation (*n* = 191).

Figure 2 shows a change in the expression of inflammation-related genes. Indeed, using the 2^−ΔΔ*C*T^ calculation method, *TNF*-α and *IL6* were slightly overexpressed in PBMCs after the 6-week *n*-3 FA supplementation (fold changes of 1.05 ± 0.38 and 1.18 ± 0.49, respectively), but relative quantification (RQ) within the −0.5 to 2.0 fold are considered as non-significant.

**Figure 3:**
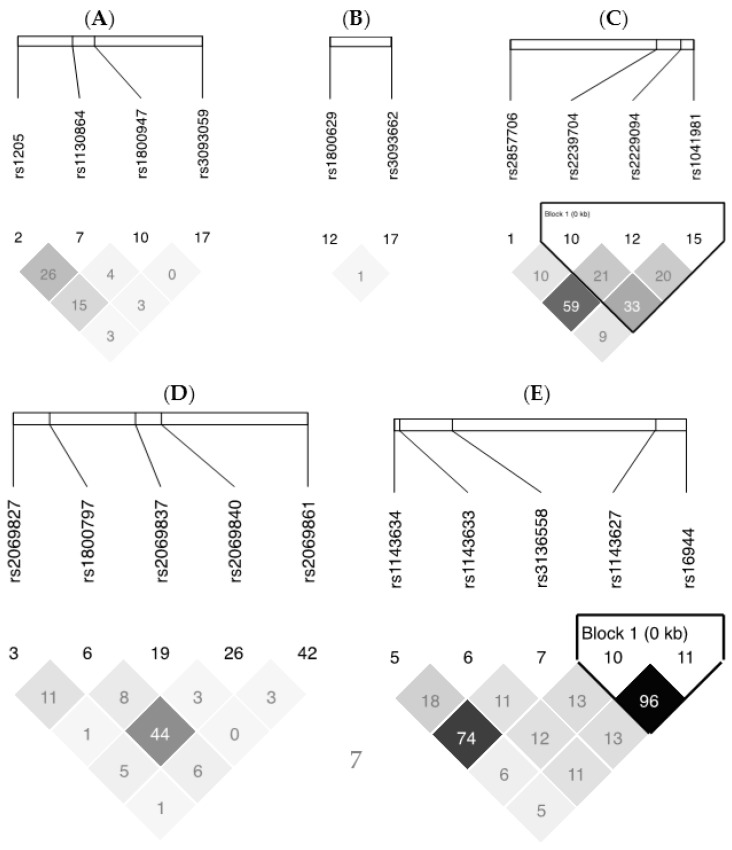
LD plots of selected inflammation-related genes. The lighter the shade of grey, the lesser is the correlation between two SNPs. (**A**) *CRP* gene; (**B**) *TNF*-α gene; (**C**) *LTA* gene; (**D**) *IL6* gene; (**E**) *IL-1*β gene.

All selected SNPs were in HWE and LD plots from Haploview v4.2 for each gene are presented in Figure 3. For SNPs within the *TNF*-*LTA* gene cluster, 6 SNPS covered 93% of the known genetic variability, for *IL6*, 5 SNPS covered 100%, for *IL-1*β, 5 SNPs covered 100%, and for *CRP*, 4 SNPs covered 100%. Table 4 reports all the selected SNPs within the five inflammation-related genes studied.

**Figure 4:**
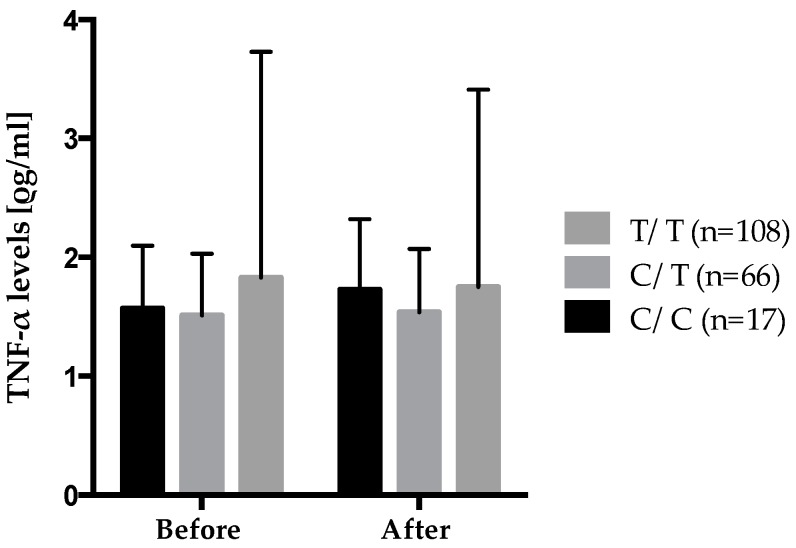
Gene–diet interaction on plasma TNF-α levels according to rs2229094 (*TNF*-α) (*n* = 191).

In a repeated MIXED model adjusted for the effects of age, sex, and BMI, several gene–diet interactions impacting inflammatory marker levels were observed following the *n*-3 FA supplementation, as shown in Table 5. Figure 4 shows the gene–diet interaction on plasma TNF-α levels according to rs2229094 where carriers of the mutated allele increased their plasma TNF-α levels after the 6-week *n*-3 FA supplementation while wild type homozygotes decreased theirs. Also, significant differences were observed in the genotype distribution of rs2229094 between positive and negative responders according to delta TNF-α levels where positive responders decreased their plasma TNF-α levels after the supplementation were an increase was observed in negative responders. There was a higher proportion of C/C HMZ that were negative responders (6.8%) *vs.* positive responders (2.1%) (*p* = 0.0003). An interaction between rs2229094 (*TNF-LTA*) and the 6-week *n*-3 FA supplementation is of particular interest because that SNP is located in an exon and is responsible for an amino acid change (Cys13Arg). Moreover, analyses with SIFT argue for potential functional effect of this SNP as the amino acid change was considered damaged using homologues in protein alignment (score = 0.04), but tolerated using orthologues in protein alignment.

## 3. Discussion

We observed in the present study that *n*-3 FAs may interact with SNPs from inflammation-related genes to modulate plasma cytokine levels. There was no reduction of plasma IL-6 and TNF-α as well as CRP levels, but several gene–diet interactions with SNPs within inflammation-related genes and *n*-3 FAs have been found potentially modulating inflammatory marker levels. These findings are consistent with the well-known anti-inflammatory properties of *n*-3 FAs, but the amplitude of the results may differ according to an individual’s genotype. In addition to nutrigenetic effects, baseline plasma EPA, DHA or total *n*-3 FA levels are negatively associated with plasma cytokine and CRP levels (Figure 1).

Ferrucci *et al.* have reported that plasma levels of *n*-3 FAs were independently associated with lower levels of pro-inflammatory markers and higher levels of anti-inflammatory markers independent of confounders such as age, sex, BMI, smoking status, education, energy intake, and potentially confounding drug treatment among others [13]. They observed that lower EPA and total *n*-3 FAs were associated with higher IL-6 and TNF-α levels (for total *n*-3 FAs only) [13]. Our results are moving in the same direction as shown by the correlations between baseline FA levels with the principal inflammatory biomarkers (Figure 1).

Trebble *et al.* have shown in 16 healthy subjects, that the dose-response relationships between *n*-3 FA, phospholipid composition and cytokine production by PBMCs is U-shaped meaning that an intermediate levels of EPA within plasma and cell membrane phospholipids, resulting from an *n*-3 FA supplementation of <2.0 g/day, may be associated with a greater inhibitory effect on TNF-α release than higher EPA concentrations resulting from *n*-3 FA supplementary intakes of >2.0 g/day [14]. Rees *et al.* have suggested that there is a threshold for an anti-inflammatory effect of EPA somewhere between 1.35 and 2.7 g/day [15]. However, in the present study, there was no significant reduction in plasma CRP, IL-6 or TNF-α levels despite the high doses of *n*-3 FAs used during the protocol (Table 4). This could be explained by low baseline levels of inflammatory markers, by the exclusion of individuals with plasma CRP levels >10.0 mg/L, or by the intermediate doses of EPA given to study participants. Moreover, there could be a differential effect of each of the *n*-3 FAs impacting inflammatory marker levels.

Moreover, besides the independent influence of dietary FAs on cytokine levels, some SNPs in the *TNF-LTA* gene family may be related to the inter-individual variability observed in plasma TNF-α levels and *TNF*-α gene expression [6]. In this study, rs2229094 (*TNF*-α*)* is associated with plasma TNF-α levels. This SNP is of particular interest due to its exonic location and the presence of a missense mutation (Cys13Arg) that is located at a conserved residue. Recent studies have shown that rs2229094 was associated with type 2 diabetes [16], CRP levels [16], sepsis [17], Crohn’s disease [18], and cancer risk [19]. According to Huang *et al.*, four functional SNPs of the LTA gene, including rs2229094, may exert possible regulatory effects on gene expression and cytokine production [19].

Another SNPs in the *TNF-LTA* gene family was associated with inflammatory responses. Indeed, we observed an interaction between rs1800629 and *n*-3 FAs modulating CRP levels where carriers of the mutated allele had significantly higher CRP levels than the wild-type genotype after fish oil supplementation (Table 5). Song *et al.* have found that the mutated allele of rs1800629 was associated with increased TNF-α production in PBMCs from healthy subjects after stimulation with LPS [20]. Studies have reported that dietary fat intake could alter the relationship between *TNF* −308G>A (also referred to as rs1800629) with adiposity and serum lipid concentrations. The main results of these studies are that *TNF* −308G>A was associated with an increased risk of obesity and dyslipidaemia, and carriers of the mutated allele appeared to be more responsive to dietary fat intake [21,22]. Meydani *et al.* reported that the production of the cytokines IL-1β, TNF-α, and IL-6 by mononuclear cells was reduced after the consumption of the low-fat, high-fish diet [23].

Evidence have shown that carriers of the mutated allele had a 2-fold increase in the *TNF*-α transcriptional activity, thus playing a role in the altered *TNF*-α gene expression possibly leading to an increase in cytokine production [24]. Moreover, Wilson *et al.* have found that rs1800629 may exert direct effects on *TNF*-α gene regulation, potentially leading to high TNF-α phenotype (expression levels, transcriptional activity, inflammatory marker levels) and more severe infection diseases in TNF2 homozygotes [25]. For example, Antonicelli *et al.* reported that carriers of *TNF*-α gene −308G>A were more likely to be affected by severe ischemic damage in a case-control study including elderly Italian individuals with and without coronary heart disease [26]. However, in this study, there were no significant differences observed in *TNF*-α gene expression nor in plasma TNF-α levels according to the rs1800629 genotype both in dominant and additive models. Although the relative change in TNF-α levels after the supplementation was not statistically significant between genotype groups owning to the small sample size, the difference was clinically relevant and seems to be in agreement with the actual literature (delta TNF-α→G/G: 28.0% *vs.* A/G: 3.3% *vs.* A/A 1.3%). This heterogeneity observed in the response to fish oil could be partly explained by the replacement of arachidonic acids by *n*-3 FAs into cell membranes leading to the production of diverse eicosanoids. Accordingly, Grimble *et al.* have suggested that the overall effect on TNF-α production (inhibition or stimulation) probably depends on the balance among the different stimulatory and inhibitory eicosanoids produced from arachidonic acid and EPA [7].

Although gene expression of inflammation-related genes is often decreased following an increase of the *n*-*3* FAs in the diet [27,28,29], several studies reported no significant decrease in plasmatic levels of inflammatory biomarkers, such as TNF-α, IL-6 or CRP [13,30]. In the present study, *TNF*-α and *IL6* genes were slightly overexpressed in PBMCs after the 6-week *n*-3 FA supplementation, but relative quantification (RQ) within the −0.5 to 2.0 fold are considered not significant. There was no clear effect of the 6-week *n*-3 FA supplementation on the expression of the two selected inflammation-related genes (*IL6* and *TNF*-α) on a metabolically healthy, but slightly overweight population, even with the use of triplicates to ensure a better reduction in biological variance. These results failed to demonstrate changes in expression levels of *TNF*-α and *IL6* when looking at these two genes specifically using a real-time PCR approach. However, Rudkowska *et al.* and Bouwens *et al.* have shown using transcriptomic approaches that inflammation-related pathways in PBMCs were changed to the anti-inflammatory direction after an *n*-3 FA supplementation [9,10].

### Strengths and Limitations

Several limitations of the present study need to be addressed. Participants were relatively young (mean age of 30.8 ± 8.7 years) and they had low inflammatory biomarker levels at baseline. The patients were healthy and we excluded participants having plasma CRP levels > 10 mg/L. We did not observe difference in gene expression of inflammation-related genes after the supplementation. This could be attributable to the use of the 2^−ΔΔ*C*T^ calculation method assuming that the endogenous control gene and target gene have both similar efficiencies. Also, this study did not allow to isolate the effect of a single FA and its potential gene–diet interactions on inflammatory markers due to the composition of the *n*-3 FA fish oil capsules given to participants that contained EPA and DHA.

## 4. Experimental Section

### 4.1. Subjects

A total of 254 unrelated subjects from the greater Quebec City metropolitan area were recruited through emails sent to University students and employees via advertisements in local newspapers. Inclusion criteria were as follow: (1) to be aged between 18 and 50 years; (2) being non-smoker; (3) having a body mass index (BMI) between 25 and 40 kg/m^2^; and (4) being free of pharmacologic lipid lowering treatment and/or metabolic disorders. Subjects who had taken *n*-3 FA supplements six months prior to the beginning of the study were excluded. A total of 210 subjects completed the supplementation protocol. Individuals with CRP levels >10 mg/L were excluded, bringing the total down to 191 eligible individuals. This experimental protocol #C09-05-030 was approved by the CHU de Quebec ethics committee on 16 September 2009. This clinical trial was registered at clinicaltrials.gov (NCT01343342).

### 4.2. Study Design and Diets

In order to minimize the intra- and inter-variability in dietary intakes, a 2-week run-in period preceded the supplementation. During this run-in period, a registered dietitian gave individual dietary instructions in order to ensure that participants were in a stable condition before the beginning of the study. Participants received recommendations by a registered dietitian in order to follow the recommendations of the *Eating Well with Canada’s Food Guide* [31]. Participants were also asked to maintain their body weight stable throughout the whole research protocol. After the run-in period, each participant received *n*-3 FA capsules in sufficient quantity for the next six weeks. They were instructed to take five capsules/d of fish oil (total of 5 g/day of fish oil), providing a total of 3.0–3.3 g of *n*-3 FAs including 1.9–2.2 g of eicosapentaenoic acid (EPA) and 1.1 g of docosahexaenoic acid (DHA). Before the run-in period, each participant completed a validated food-frequency questionnaire (FFQ) [32] supervised by a registered dietitian. This 91-item FFQ is based on typical food items found in North America. Moreover, they were asked to complete two 3-day food records—prior to and after the *n*-3 FA supplementation period. Dietary intakes data were analyzed using Nutrition Data system for Research software v.2011 (Nutrition Coordinating Center (NCC), University of Minnesota, Minneapolis, MN, USA).

### 4.3. Anthropometric Measurements

Body weight, height and waist circumference were measured at every visit in accordance with the Airlie Conference on the Standardization of anthropometric measurements [33]. BMI was calculated as weight per meter squared (kg/m^2^).

### 4.4. Biochemical Parameters

Because the study design was not intended to look specifically at inflammation, only a small subset of inflammatory biomarkers were available for this reanalysis. Blood samples were collected after a 12 h overnight fast and 48h alcohol abstinence, from an antecubital vein into vacutainer tubes containing EDTA. Plasma CRP was measured by nephelometry (Dade Behring, Deerfield, IL, USA) using a sensitive assay, as described previously [34]. Plasma concentrations of IL6 and TNF-α were measured with high-sensitivity ELISA kits including: Human IL6 Quantikine HS ELISA Kit Minneapolis, MN, USA (R & D Systems, Minneapolis, MN, USA (HS600B)) and Human TNF-α Quantikine HS ELISA Kit (R & D Systems (HSTA00D)) [34].

### 4.5. Measurement of FA Composition in Plasma Phospholipids

FA composition of plasma phospholipids was determined by gas chromatography. Venous blood was drawn into EDTA tubes, then separated by centrifugation at 500 *g* for 6 min and stored at −80 °C for subsequent analyses. Plasma lipids were extracted with chloroform:methanol (2:1, by volume) according to a modified Folch method [35]. Total phospholipids were then isolated with isopropyl ether:acetic acid (96:4) by thin layer chromatography [36]. Isolated plasma phospholipids were then methylated [37]. FA profiles were obtained after methylation in methanol/benzene 4:1 (*v*/*v*) [37] and capillary gas chromatography using a temperature gradient on a HP5890 gas chromatograph (Hewlett Packard, Toronto, ON, Canada) equipped with a HP-88 capillary column (100 m × 0.25 mm i.d. × 0.20 µm film thickness; Agilent Technologies, Palo Atto, CA, USA) coupled with a flame ionization detector. Helium was used as carrier gas using a split ratio of 1:80). FA were identified according to their retention time as well as the following methylated FAs C22:5*n*-6 (Larodan AB, Malmö, Sweden) and C22:5*n*-3 (Supelco Inc., Bellefonte, PA, USA). Finally, phospholipids FA profiles were determined using the relative percentage areas of total FAs.

### 4.6. SNP Selection and Genotyping

SNPs in the *TNF-LTA gene cluster* (6 SNPs)*, IL6* (5 SNPs) and *CRP* (4 SNPs) were identified using the International HapMap Project SNP database, based on the National Center for Biotechnology Information (NCBI) B36 assembly Data Rel 28. phase II + III, build 126. The *TNF-LTA* gene cluster is made of two genes that are located very close to each other on chromosome 6 (*LTA* location: Chr6p21.3, 31,572,099 … 31,574,324; *TNF*-α location: Chr6p21.3, 31,575,567 … 31,578,336). *LTA* is also referred to as member 1 of the *TNF*-superfamily. Five hundred kilo-base pairs (kbp) downstream of each gene and 2500 kbp upstream of each gene were added to cover the 5′UTR and 3′UTR regions. Gene Tagger procedure in Haploview v4.2 was used to determine SNPs using a minor allele frequency (MAF) ≥ 5% and pairwise tagging (*r*^2^ ≥ 0.8). Subsequently, the linkage disequilibrium (LD) was examined for all SNPs using the LD Plot procedure in Haploview v4.2. The SIGMA GenElute Gel Extraction Kit (Sigma-Aldrich Co., St. Louis, MO, USA) has been used to extract genomic DNA. Selected SNPs of the *TNF-LTA* gene family (rs1041981, rs2857706, rs1800629, rs2239704, rs3093662, and rs2229094), *IL6* (rs2069861, rs2069840, rs2069837, rs2069827, and rs1800797), *IL1*β (rs1143633, rs1143634, rs16944, rs3136558, rs1143627) and *CRP* (rs1800947, rs3093059, rs1130864, and rs1205) have been genotyped using validated primers and TaqMan probes (Thermo Fisher Scientific, Waltham, MA, USA) [38]. DNA was mixed with TaqMan Universal PCR Master Mix (Thermo Fisher Scientific), with a gene-specific primer and with probe mixture (pre-developed TaqMan SNP Genotyping Assays; Thermo Fisher Scientific) in a final volume of 10 μL. Genotypes were assessed using a 7500 RT-PCR System and the ABI Prism Sequence Detection System v2.0.5 was used to analyse the data (Thermo Fisher Scientific).

### 4.7. Gene Expression

Gene expression of *IL6* and *TNF*-α genes was measured following the 6-week *n*-3 FA supplementation. Complementary DNA (cDNA) was mixed with TaqMan Universal PCR Master Mix (Thermo Fisher Scientific) and a gene-specific primer and probe mixture in a final volume of 20 μL. All samples were run on a 7500 Fast Real Time PCR System (Thermo Fisher Scientific) using the following thermal cycling profile: 50 °C (2 min), 95 °C (10 min), followed by 40 steps of 95 °C for 15 s and 60 °C for 60 s. The real-time PCR data were imported into Expression Suite Software v1.0. These samples were analysed in triplicate and calibrated to the *GAPDH* gene (endogenous control; *GAPDH*: Hs99999905_ml). Relative quantification estimations were achieved using the 2^−ΔΔ*C*T^ calculation method [39].

### 4.8. Statistical Analyses

This study is a reanalysis of the Fatty Acid Sensor (FAS) Study that primarily wanted to understand how genes and environment act together on the cardiometabolic risk profile. Data were analyzed with SAS Genetics v9.3. Values that were not normally distributed were log_(10)_-transformed, negative reciprocal or normalised by a Box-Cox transformation (TNF-α) before analysis. Subjects were categorised as positive (Δ < 0%) or negative (Δ ≥ 0%) responders based on their relative change of plasma TNF-α, CRP or IL-6 levels after the supplementation. The MIXED procedure for repeated measures was used to test for significant differences in metabolic characteristics between men and women at baseline and for differences between various nutrient intakes prior to and after *n*-3 FA supplementation and to test for the effects of the genotype, the supplementation and the genotype by supplementation (time) interaction on inflammatory marker levels when age, sex and BMI were included in the model. Partial Spearman correlations were calculated between EPA, DHA or total *n*-3 of plasma phospholipids (in % of total FAs) and inflammatory markers. The ALLELE procedure was used to verify the departure from Hardy-Weinberg equilibrium (HWE) and to calculate the minor allele frequency. Individuals with CRP levels > 10 mg/L were excluded bringing the total to 191 eligible individuals. SIFT web-based software was used to predict the effect of amino acid substitution and all tests were run under default threshold values. Finally, the FREQ procedure was used to verify differences in genotype frequency distribution between positive and negative responders to the *n*-3 FA supplementation. Statistical significance was defined as *p* ≤ 0.05.

## 5. Conclusions

Overall, this study shows that a 6-week *n*-3 FA supplementation with 5 g of fish oil daily did not alter gene expression levels of *TNF*-α and *IL6* in PBMCs and did not have an impact on inflammatory biomarker levels. However, significant gene–diet interactions were observed between SNPs within inflammation-related genes modulating plasma inflammatory biomarker levels. These gene–diet interactions may potentially explain the large inter-individual variability observed in plasma inflammatory response following an *n*-3 FA supplementation.

## Figures and Tables

**Table 1:** Characteristics of the study participants at baseline (*n* = 191).

Characteristics	All (*n* = 191)	Men (*n* = 95, 49.7%)	Women (*n* = 96, 50.3%)
Age	30.8 ± 8.7	30.9 ± 8.1	30.8 ± 9.3
Waist circumference, cm	93.2 ± 10.6	94.8 ± 10.9	91.6 ± 10.1
BMI, kg/m^2^	27.2 ± 3.6	27.5 ± 3.6	27.9 ± 3.5

Values are means ± SD.

**Table 2:** Pre- and post-supplementation inflammatory marker levels (*n* = 191).

Biomarkers	Pre *n*-3 FA	Post *n*-3 FA	*p*-Value ^1^
CRP levels (mg/L) ^2^	1.78 ± 2.09	1.81 ± 2.07	0.95
TNF-α levels (ρg/mL) ^3^	1.70 ± 1.48	1.68 ± 1.30	0.69
IL-6 levels, (ρg/mL) ^2^	1.34 ± 1.13	1.28 ± 0.98	0.54

Values are means ± SD. * *p* < 0.05; ^1^ *p*-value are derived from a MIXED procedure for repeated measures adjusted for age, sex and BMI (except for the BMI and the waist circumference that were adjusted only for age and sex); ^2^ values were log_(10)_ transformed; ^3^ values were normalised using the Box-Cox transformation.

**Table 3:** Dietary intakes pre- and post-supplementation with *n*-3 PUFAs (*n* = 191).

Dietary Intakes	Pre-Suppl.	Post-Suppl.	*p-*Value ^1^
		(Including *n*-3 FA Supplements)	
Energy, (kcal)	2290 ± 599	2196 ± 570	0.003
Carbohydrate, (% of TEI)	50.5 ± 7.2	48.5 ± 7.8	0.001
Carbohydrate, (g/day)	288.9 ± 79.8	264.1 ± 78.1	<0.0001
Protein, (% of TEI)	17.4 ± 3.3	16.9 ± 3.1	0.15
Protein, (g/day)	98.6 ± 30.6	93.2 ± 30.1	0.002
Total fat, (% of TEI)	32.5 ± 6.0	35.2 ± 6.3	<0.0001
Total fat, (g/day)	85.0 ± 29.7	86.8 ± 29.9	0.56
SFA, (% of TEI)	11.1 ± 3.1	10.3 ± 3.0	0.001
SFA, (g/day)	29.1 ± 12.1	25.4 ± 10.5	<0.0001
MUFA, (% of TEI)	11.8 ± 2.8	12.0 ± 3.3	0.41
MUFA, (g/day)	30.8 ± 11.8	29.7 ± 12.5	0.17
PUFA, (% of TEI)	5.9 ± 2.1	6.9 ± 2.1	<0.0001
PUFA, (g/day)	15.3 ± 6.7	16.9 ± 6.7	0.001

^1^ *p*-value provided by a paired *t*-test. TEI stands for “Total energy intakes”; SFA stands for “Saturated fat”.

**Table 4:** Selected SNPs within *TNF-LTA*, *IL-6*, *IL-1*β, and *CRP* genes.

Genes	dbSNP No. ^a^	Sequence ^b^	Position	AA	CC	CA	CG	CT	GA	GG	GT	AT	TT
				*n* (%)									
*TNF-LTA*	rs1041981	CA[A/C]CC	Missense [Thr]→[Asn]	22 (10.5)	97 (46.2)	91 (43.3)							
*TNF-LTA*	rs2857706	TA[A/G]GT	Intron		151 (7.2)			52 (24.8)					7 (3.3)
*TNF-LTA*	rs1800629	TG[A/G]GG	nearGene-5	2 (1.0)					45 (21.4)	163 (77.6)			
*TNF-LTA*	rs2239704	GC[G/T]GG	UTR-5	49 (23.3)	72 (34.3)	89 (42.4)							
*TNF-LTA*	rs3093662	AC[A/G]GA	Intron	178 (84.8)					29 (13.8)	3 (1.4)			
*TNF-LTA*	rs2229094	TG[C/T]GT	Missense [Cys]→[Arg]		19 (9.1)			74 (35.2)					117 (55.7)
*IL6*	rs2069861	AA[C/T]AA	nearGene-3		177 (84.3)			33 (15.7)					0 (0.0)
*IL6*	rs2069840	AA[C/G]TT	Intron		95 (45.2)		92 (43.8)			23 (11.0)			
*IL6*	rs2069837	TA[A/G]AT	Intron	181 (86.2)					28 (13.3)	1 (0.5)			
*IL6*	rs2069827	TC[G/T]AT	nearGene-5							172 (81.9)	38 (18.1)		0 (0.0)
*IL6*	rs1800797	GG[A/G]TG	nearGene-5	73 (34.8)					111 (52.9)	26 (12.4)			
*IL1B*	rs1143634	TT[C/T]GA	Cds-synon [Phe]→[Phe]		129 (61.4)			70 (33.3)					11 (5.2)
*IL1B*	rs1143633	CC[A/G]CC	Intron	32 (15.2)					94 (44.8)	84 (40.0)			
*IL1B*	rs16944	TC[A/G]GG	nearGene-5	28 (13.3)					82 (39.1)	100 (47.6)			
*IL1B*	rs3136558	GA[C/T]CT	Intron		8 (3.8)			77 (36.7)					125 (59.5)
*IL1B*	rs1143627	GC[C/T]AT	nearGene-5		29 (13.8)			81 (38.6)					100 (47.6)
*CRP*	rs1800947	CT[C/G]TC	Cds-synon [Leu]→[Leu]		190 (90.5)		20 (9.5)						0 (0.0)
*CRP*	rs3093059	AT[C/T]GG	nearGene-5		2 (1.0)			32 (15.2)					176 (83.6)
*CRP*	rs1130864	AA[C/T]GG	UTR-3		102 (48.6)			84 (40.0)					24 (11.4)
*CRP*	rs1205	CA[C/T]AG	UTR-3		99 (47.1)			89 (42.4)					22 (10.5)

**^a^** dbSNP No. from HapMap Data Rel 28 Phase II + III, 10 August on NCBI b36 Assembly dbSNP b126 database; **^b^** Genes sequences from dbSNP short genetics variations NCBI reference assembly.

**Table 5:** Gene–diet interaction on inflammatory markers levels.

Gene	Biomarker	SNPs	Genotype by Suppl.	β ± SE	*p*-Value for the Genotype	*p*-Value for the *n*-3 Suppl.	*p*-Value for the Interaction
*IL-1*β	IL-6 levels (ρg/mL)	rs1143627	CC × *n*-3 suppl.	−0.014 ± 0.025	0.91	0.78	0.02
			CT × *n*-3 suppl.	0.037 ± 0.014			
			TT × *n*-3 suppl.	−0.014 ± 0.013			
	IL-6 levels (ρg/mL)	rs16944	AA × *n*-3 suppl.	−0.018 ± 0.025	0.87	0.87	0.02
			AG × *n*-3 suppl.	0.038 ± 0.014			
			GG × *n*-3 suppl.	−0.014 ± 0.013			
*IL6*	IL-6 levels (ρg/mL)	rs1800797	AA × *n*-3 suppl.	0.025 ± 0.015	0.30	0.64	0.05
			AG × *n*-3 suppl.	−0.023 ± 0.013			
			GG × *n*-3 suppl.	−0.016 ± 0.025			
	IL-6 levels (ρg/mL)	rs2069840	CC × *n*-3 suppl.	−0.010 ± 0.013	0.11	0.28	0.04
			CG × *n*-3 suppl.	−0.016 ± 0.014			
			GG × *n*-3 suppl.	0.063 ± 0.029			
*TNF-LTA gene family*	TNF-α levels, (ρg/mL)	rs2229094	CC × *n*-3 suppl.	0.022 ± 0.012	0.85	0.20	0.03
			CT × *n*-3 suppl.	0.005 ± 0.006			
			TT × *n*-3 suppl.	−0.009 ± 0.005			
	CRP levels (mg/L)	rs1800629	GG × *n*-3 suppl.	−0.012 ± 0.016	0.37	0.17	0.04
			[AA+GA] × *n*-3 suppl.	0.058 ± 0.030			

β: regression coefficient. The MIXED model for repeated measures included the SNP, the time and the interaction term with adjustments for the effects of age, sex and BMI.

## Abbreviations

The following abbreviations are used in this manuscript:

*n*-3	omega-3
FAs	fatty acids
SNPs	single nucleotide polymorphisms
TNF-α	tumor necrosis factor alpha
IL6	interleukin 6
CRP	C-reactive protein
PBMCs	peripheral blood mononuclear cells
BMI	body mass index
EPA	eicosapentaenoic acid
DHA	docosahexaenoic acid
FFQ	food-frequency questionnaire
TG	triglycerides
MAF	minor allele frequency
HWE	Hardy-Weinberg equilibrium
